# DDX1 from Cherry valley duck mediates signaling pathways and anti-NDRV activity

**DOI:** 10.1186/s13567-020-00889-4

**Published:** 2021-01-20

**Authors:** Huihui Zhang, Xingdong Song, Tianxu Li, Jinchao Wang, Bin Xing, Xinyu Zhai, Jinjian Luo, Xiaofang Hu, Xiaolan Hou, Liangmeng Wei

**Affiliations:** 1 Sino-German Cooperative Research Centre for Zoonosis of Animal Origin of Shandong Province, Shandong Provincial Key Laboratory of Animal Biotechnology and Disease Control and Prevention, Shandong Provincial Engineering Technology Research Center of Animal Disease Control and Prevention, College of Animal Science and Veterinary Medicine, Shandong Agricultural University, 61 Daizong Road, Tai’an, 271018 Shandong Province China; 2 Collaborative Innovation Center for the Origin and Control of Emerging Infectious Diseases, College of Basic Medical Sciences, Shandong First Medical University, Tai’an, 271000 Shandong Province China

## Abstract

Novel duck reovirus (NDRV) causes severe economic losses to the duck industry, which is characterized by hemorrhagic spots and necrotic foci of the livers and spleens. DEAD-box helicase 1 (DDX1) plays a critical role in the innate immune system against viral infection. However, the role of duck DDX1 (duDDX1) in anti-RNA virus infection, especially in the anti-NDRV infection, has yet to be elucidated. In the present study, the full-length cDNA of duDDX1 (2223 bp encode 740 amino acids) was firstly cloned from the spleen of healthy Cherry valley ducks, and the phylogenetic tree indicated that the duDDX1 has the closest relationship with Anas platyrhynchos in the bird branch. The duDDX1 mRNA was widely distributed in all tested tissues, especially in the duodenum, liver, and spleen. Overexpression of duDDX1 in primary duck embryo fibroblast (DEF) cells triggered the activation of transcription factors IRF-7 and NF-κB, as well as IFN-β expression, and the expression of the Toll-like receptors (TLR2, TLR3, and TLR4) was significantly increased. Importantly, after overexpressing or knocking down duDDX1 and infecting NDRV in DEF cells, duDDX1 inhibits the replication of NDRV virus and also regulates the expression of pattern recognition receptors and cytokines. This indicates that duDDX1 may play an important role in the innate immune response of ducks to NDRV. Collectively, we first cloned DDX1 from ducks and analyzed its biological functions. Secondly, we proved that duck DDX1 participates in anti-NDRV infection, and innovated new ideas for the prevention and control of duck virus infection.

## Introduction

The new duck reovirus (NDRV) is a double-stranded RNA (dsRNA) virus and a member of the Orthoreovirus genus in the Reoviridae family. The genome of the virus consists of 10 genome segments, L1-3, M1-3 and S1-4 [1]. The encoded NDRV S1 segment is similar to avian reovirus (ARV) [2], with three partially overlapping open reading frames (ORFs), encoding p10, p18, and σC, respectively [3]. Among them, σC can produce specific neutralizing antibodies, and p17 protein can inhibit the host immune response and affect the regulatory response of interferon.

In the process of virus replication, first, the virus-encoded dsRNA-dependent RNA polymerase uses the negative-strand RNA of the viral genome as a template to synthesize mRNA. Subsequently, the viral mRNA completes the capping reaction when it leaves the viral core through the channel formed by the capping enzyme λC [4]. Finally, viral mRNA plays a dual function in the infected cell, synthesizing viral proteins in the ribosome and as a template to synthesize viral genome negative-strand RNA.

NDRV infection mainly causes hemorrhage and necrosis of the liver and spleen, and may also lead to the transfer of intestinal flora1 [5], which is different from diseases caused by Muscovy duck reovirus (MDRV) [6]. Since it was first discovered in China in 2005, it has caused serious economic losses in the duck industry. Although there are many reports about the identification and diagnosis of NDRV [6–8], there are few reports about NDRV inducing host antiviral natural immune response.

Pattern recognition receptors (PRRs) are located on multiple types of innate immune cells, and can initiate immune responses to specific pathogen-associated molecular patterns (PAMPs) exclusively present on microbes (such as viruses, bacteria, parasites, and fungi) [9, 10]. PRRs include the Toll-like receptors (TLRs), the retinoic acid-inducble gene I-like receptors (RLRs), the nucleotide oligomerization domain-like receptors (NLRs) and cytosolic DNA sensors [11, 12]. Once PAMPs were recognized, the signal triggered by PRRs will activate downstream signaling pathways to induce type I interferon (IFN-I) and other pro-inflammatory cytokines, thereby establishing an antiviral immune system [13, 14]. IFN-I mainly inhibits the replication and spread of the virus by producing Interferon-stimulated genes (ISGs) [15].

DEAD-box helicases belong to the helicase superfamily 2 (SF2) and share at least eight conserved sequence motifs (motifs I, Ia, Ib, II, III, IV, V, and VI) spread, and are named DEAD-box helicases because they contain Asp-Glu-Ala-Asp (DEAD) [16]. The DEAD-box helicases core consist of two RecA-related domains. In addition to the helicase core, DEAD-box helicases usually also contains N and C-terminal extensions that provide specificity for RNA and/or protein interactions [17]. DEAD-box helicases play crucial roles in RNA metabolism such as splicing, ribosome biogenesis, RNA transport, degradation and translation [18].

DEAD-box helicase 1(DDX1), a member of the DEAD-box RNA helicases family, and was first discovered in retinoblastoma and neuroblastoma in 1993 [19]. Previous experiments proved that DDX1 can play a role in cell proliferation and RNA metabolism of differentiated cells, especially to elevate the level of DDX1 in neuroectoder [20]. DDX1 can bind to the subunit of NF-κB RelA, and enhance NF-κB-mediated gene expression [21]. In addition, DDX1 can participate in the transfer of RNA between the cytoplasm and the nucleus and the cleavage of tRNA [22, 23], and DDX1 is a cofactor of the miRNA cleavage processing enzyme Drosha, which can regulate the post-transcriptional maturation of miRNA [23]. DDX1 can also regulate insulin translation after binding to insulin Mrna [24]. It suggesting that DDX1 is involved in multiple cellular processes of RNA metabolism.

Although DDX1 has been studied in human [25], chicken [20] and mice [26], it has not been found in ducks. In this study, we cloned the duck-derived DDX1 from Cherry valley duck spleen, analyzed its biological characteristics and its expression in various tissues, and studied the role of duDDX1 in RNA viruses, especially in NDRV infections. It is expected to provide a reference for the in-depth study of duDDX1 in the way to regulate the host’s innate immune mechanism.

## Materials and methods

### Animals, cells, and virus

Cherry valley ducks were purchased from a farm in Tai'an, China. Primary duck embryo fibroblast (DEF) cells were extracted from 10-day-old duck embryos and cultured in DMEM (GIBCO, Grand Island, NY, USA) with 10% fetal bovine serum (GIBCO, Grand Island, NY, USA). DEF cells were incubated at 37 ℃ in a 5% CO_2_ incubator. New duck reovirus (NDRV) was previously preserved in the laboratory [27].

### Cloning of duDDX1

To clone the duck DDX1 (duDDX1), primers DDX1-F and DDX1-R (Table 1) were designed based on the predicted duck sequence from the National Center for Biotechnology Information (NCBI). Total RNA was extracted from duck spleen via TransZol up (Vazyme, Nan jing, China) and reverse transcription into cDNA using a HiScript^®^ II Q RT SuperMix for qPCR (Vazyme, Nan jing, China).

**Table 1:** **Primer information table of this research.**

Primer name	Sequence (5′–3′)	Purpose
JDDX1-F	CACCCTAAAGCGGCATCTCT	Gene cloning
JDDX1-R	TCAAGCCTTGTCTCAAGTGTG	Gene cloning
gDDX1-F	CTTGGTACCGAGCTCGGATCCGCCACCATGGCGGCGTTCTCGGAAATGGGTGTTATGC	Gene cloning
gDDX1-R	CTCTAGACTCGAGCGGCCGCTCAGAATGTTCTGAACAGCT	Gene cloning
qDDX1-F	GAACCTTCCAAATGCTCCAA	qRT-PCR
qDDX1-R	AGCTTCATCCAGAACGAGGA	qRT-PCR
qIFN-α-F	TCCTCCAACACCTCTTCGAC	qRT-PCR
qIFN-α-R	GGGCTGTAGGTGTGGTTCTG	qRT-PCR
qIFN-β-F	AGATGGCTCCCAGCTCTACA	qRT-PCR
qIFN-β-R	AGTGGTTGAGCTGGTTGAGG	qRT-PCR
qIFN-γ-F	GCTGATGGCAATCCTGTTTT	qRT-PCR
qIFN-γ-R	GGATTTTCAAGCCAGTCAGC	qRT-PCR
qPKR-F	AATTCCTTGCCTTTTCATTCAA	qRT-PCR
qPKR-R	TTTGTTTTGTGCCATATCTTGG	qRT-PCR
qMx-F	TGCTGTCCTTCATGACTTCG	qRT-PCR
qMx-R	GCTTTGCTGAGCCGATTAAC	qRT-PCR
qOAS-F	TCTTCCTCAGCTGCTTCTCC	qRT-PCR
qOAS-R	ACTTCGATGGACTCGCTGTT	qRT-PCR
qIL-1β-F	TCATCTTCTACCGCCTGGAC	qRT-PCR
qIL-1β-R	GTAGGTGGCGATGTTGACCT	qRT-PCR
qIL-6-F	TTCGACGAGGAGAAATGCTT	qRT-PCR
qIL-6-R	CCTTATCGTCGTTGCCAGAT	qRT-PCR
qIL-8-F	AAGTTCATCCACCCTAAATC	qRT-PCR
qIL-8-R	GCATCAGAATTGAGCTGAGC	qRT-PCR
qMDA5-F	GCTACAGAAGATAGAAGTGTCA	qRT-PCR
qMDA5-R	CAGGATCAGATCTGGTTCAG	qRT-PCR
qRIG-I-F	GCTACCGCCGCTACATCGAG	qRT-PCR
qRIG-I-R	TGCCAGTCCTGTGTAACCTG	qRT-PCR
qTLR-2-F	AAGAAAATGGAGCTGCTGGA	qRT-PCR
qTLR-2-R	GAAAAACACAGCGCAGATCA	qRT-PCR
qTLR-3-F	GAGTTTCACACAGGATGTTTAC	qRT-PCR
qTLR-3-R	GTGAGATTTGTTCCTTGCAG	qRT-PCR
qTLR-4-F	ACCCATTGTCACCAACATCATC	qRT-PCR
qTLR-4-R	TGCCTCAGCAAGGTCTTATTCA	qRT-PCR
qNDRV-F	TGAGTGGCTGGGAACTGT	qRT-PCR
qNDRV-R	CCATAAAGGAAGCAGAAG	qRT-PCR
qGAPDH-F	ATGTTCGTGATGGGTGTGAA	qRT-PCR
qGAPDH-R	CTGTCTTCGTGTGTGGCTGT	qRT-PCR

### Biological process analysis of duDDX1

Amino acid sequences deduced from the nucleotide sequences were aligned using Clustal X and edited with DNAMAN. The structure domain of duDDX1 is identified via SMART program. Sequence homology analysis was conducted using MegAlign (DNAstar, USA). The phylogenetic tree was constructed based on DDX1 from 15 different species, including birds, fishes, and mammals.

### Animal experiments

Three health ducks (aged 3 weeks) were killed and their tissues including the heart, liver, spleen, lung, kidney, brain, cerebellum, muscle, cecum, ileum, jejunum, duodenum, windpipe, bursal of fabricius, muscular stomach, glandular, stomach, skin, esophagus, pancreas, and brainstem were collected [28]. Twenty-eight health ducks (aged 3 weeks) were randomly divided into two groups, one group was used for an infection experiment in which ducks were intramuscularly inoculated with 0.5 mL virus stocks containing 1.0 × 10^4.5^ TCID_50_. The other group was set as the control group and received an intramuscular injection of 0.5 mL sterile PBS [29]. On 1, 3, and 5 days post-infection (dpi), three ducks from each group were killed and their liver and spleen were collected. Total RNA was extracted for duDDX1 mRNA expression detection from these tissues.

### Plasmid construction

The expression construct pcDNA3.0-duDDX1-Flag was constructed by inserting full-length duDDX1 into the *Bam*H-I and *Not*-I sites of the pcDNA3.0-Flag vector using Hieff Clone™ Multi One Step Cloning Kit (Yeasen, Shanghai, China). The primers described in Table 1.

### Quantitative real-time PCR

Quantitative real-time PCR (qRT-PCR) was conducted via indicated primers (Table 1) using the LightCycler^®^ 96 SW 1.1 real-time PCR system. The qRT-PCR was performed using ChamQ^™^ SYBR qPCR Master Mix (Vazyme, Nanjing, China), and performed at a reaction volume of 20 μl according to the manufacturer's instructions. The PCR cycling conditions were: 1 cycle at 95 °C for 300 s, 40 cycles of denaturation at 95 °C for 10 s, 60 °C for 34 s, and 97 °C for 1 s. The relative expression levels of the tested mRNAs were determined using GAPDH as an internal reference using the comparative Ct (2^−ΔΔCT^) method.

### Western blotting

DEF cells were cultured in a 6-well plate. When the cells reached approximately 95% confluence, the pcDNA3.0-duDDX1-Flag or empty vector were transfected into the DEF cells, according to the instructions of Nulen Trans^™^ Liposomal Transfection Reagent (NULEN BIOTECH, Shanghai, China). At 48 h post-transfection (hpt), cells were lysed with a cell lysis buffer containing Protease inhibitor cocktail (Beyotime, Shanghai, China), and cell proteins were collected. After SDS-PAGE electrophoresis and wet transfer, the target protein was transferred to polyvinylidene fluoride (PVDF) membrane (Solarbio, Beijing, China). Seal with 5% skimmed milk powder for 1 h, and then they were incubated with mouse anti-Flag antibody (Cell Signaling Technology, Shanghai, China) and Goat Anti-Mouse IgG, HRP Conjugated (CWBIO, Beijing, China). After membrane washing, the protein bands were observed with ECL kit (BIO-Rad), and images were collected using the Tanon 5200 imaging system (Tanon, Shanghai, China).

### Dual luciferase assay

DEF cells were seeded in 24-well plates and cultured until the cells reached approximately 95% confluence, and then co-transfected with the pcDNA3.0-duDDX1-Flag or empty vector (500 ng/well), reporter plasmid (100 ng/well), and pRL-TK plasmid (50 ng/well) by Nulen Trans^™^ Liposomal Transfection Reagent (NULEN BIOTECH, Shanghai, China). Cell extracts were collected at the indicated time points and luciferase activity was measured with a dual-specific luciferase assay kit (Promega). All reporter assays were independently repeated at least three times.

### RNA interference

Three interfering RNA-targeting duDDX1 sequences were purchased from GenePharma (Shanghai, China). According to the instructions of Lipofectamine 2000 (Invitrogen, Carlsbad, CA, USA), three interfering RNAs and negative control (NC) were transfected into DEF cells in a 6-well plate. Their interference efficiency were analyzed by qRT-PCR after 36 hpt. The siRNA sequences were listed in Table 2.

**Table 2:** **The sequences of pSi-RNA.**

pSi-RNA	Sequence (5′-3′)	Positions
pSi-NC (sense)	UUCUCCGAACGUGUCACGUTT	
pSi-NC (antisense)	ACGUGACACGUUAGAATT	
pSi-duDDX1-1 (sense)	GCUUCUUCCUACAGAUAUUTT	66
pSi-duDDX1-1 (antisense)	AAUAUCUGUAGGAAGAAGCTT	
pSi-duDDX1-2 (sense)	GCAACUAGAGGCGUGACUATT	370
pSi-duDDX1-2 (antisense)	UAGUCACGCCUCUAGUUGCTT	
pSi-duDDX1-3 (sense)	GGAUGAAGCUGAUGGCCUUTT	1107
pSi-duDDX1-3 (antisense)	AAGGCCAUCAGCUUCAUCCTT	

### Virus infection

DEF cells were transfected with pcDNA3.0-duDDX1-Flag or empty vector, after 24 hpt, the transfected cells were washed twice and infected with 10 TCID_50_ NDRV for 1 h. Or DEF cells were transfected with Si-DDX1 or Si-NC, after 36 hpt, the transfected cells were washed twice and infected with 1 TCID_50_ NDRV for 1 h. The virus solution was discarded after the designated time, and add 2 mL of low serum medium (DMEM containing 2% fetal bovine serum) into each well to maintain the cells [27].

### Statistical analysis

All data were expressed as mean ± SE of three independent experiments. Significance was determined with the student *t* tests using SPSS software version 17.0 (SPSS Inc., Chicago, IL, USA). *P* < 0.05 were considered indicative of statistical significance.

### Results

#### Cloning and sequence analysis of duck DDX1

**Figure 1:**
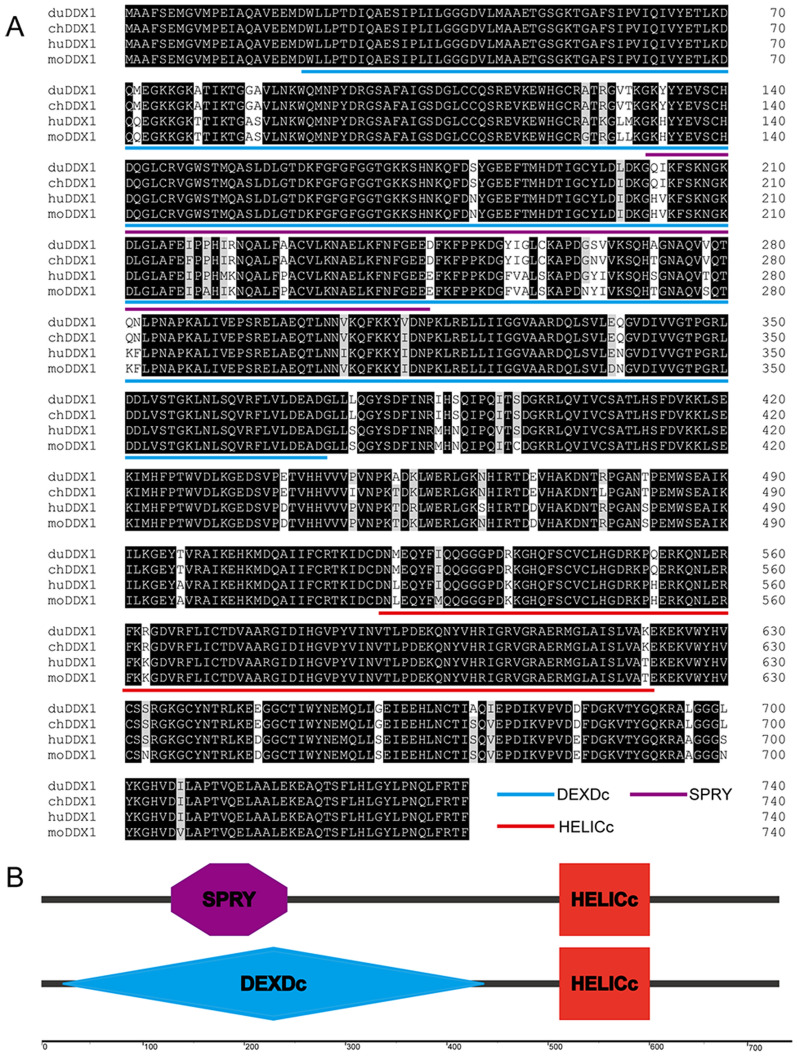
**Characterization of duDDX1.** **A** Amino-acid alignment of duDDX1. Alignment was performed using the Clustal X program and edited with DNAMAN. DDX1 sequences are shown for the Cherry valley duck (du), chicken (ch), human (hu), and mouse (mo). Black shading indicates amino acid identity, gray shading indicates similarity (50% threshold). **B** Prediction of protein domains by the SMART program.

**Figure 2:**
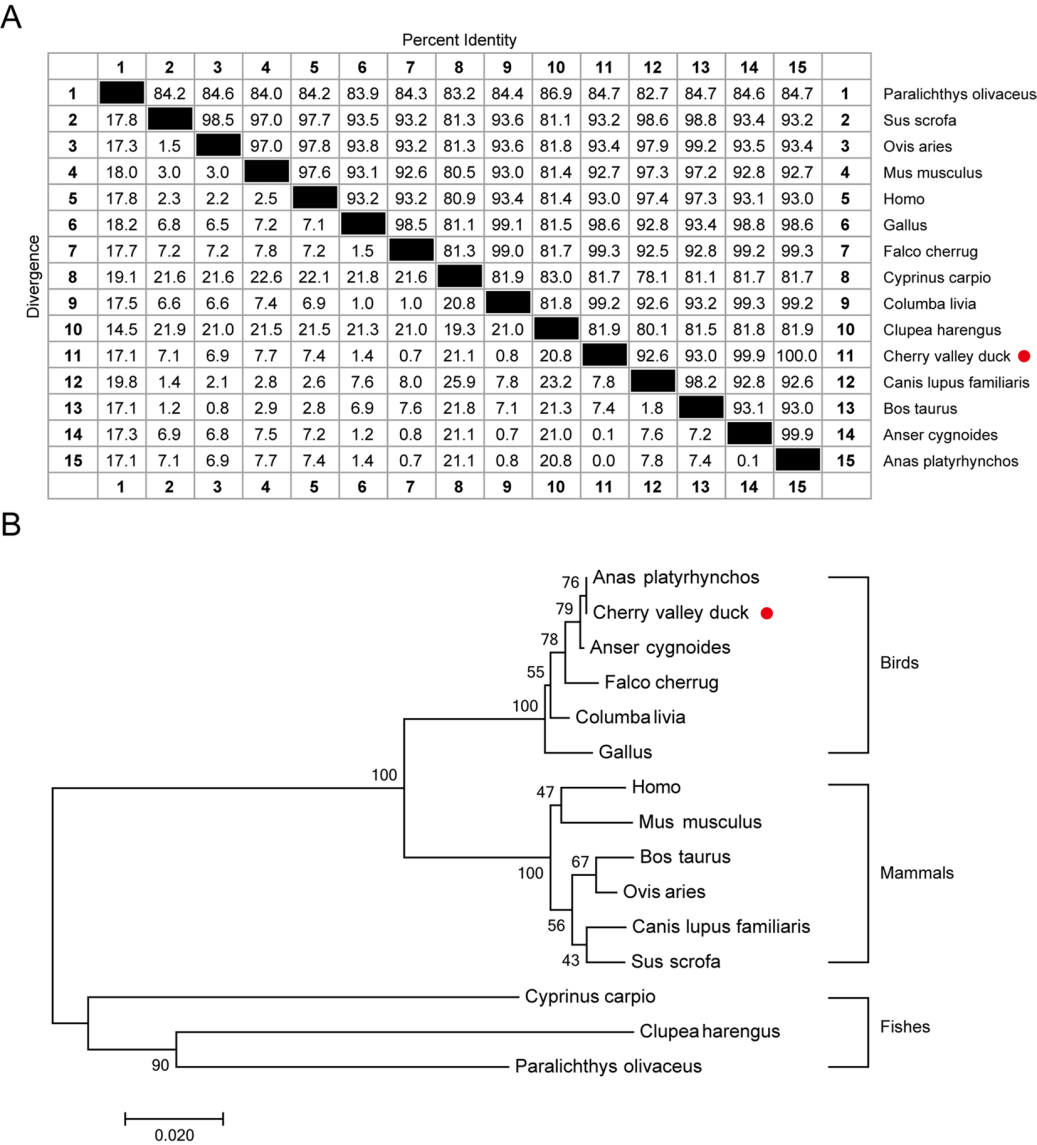
**Sequence similarity and phylogenetic analysis of duDDX1.** **A** Sequence similarity analysis of DDX1 among different species. The program was performed using MegAlign software. **B** A phylogenic tree based on duDDX1 the amino acid sequences of the Cherry valley duck and other species. The neighbor-joining tree was generated using MEGA 7.0 [37], and a 1000-replicate bootstrap analysis was performed. Scale bar is 0.02. GenBank accession numbers are shown in Table 3.

The full-length cDNA of duDDX1 contains 2223 bp (GenBank accession number, MT978184) encoded 740 amino acid residues (Figure 1A). Using SMART software to analyze the protein domains of duDDX1, it was found that duDDX1 consists of a DEXDc domain (AA 21–444), a HELICc domain (AA 520–610), and a SPRY domain (AA 130–246) (Figure 1B). Multiple sequence comparisons indicated that duDDX1 showed relatively high identity with the DDX1 of chicken (98.6%), with 93.2% and 93% identity to pig and human DDX1 proteins, respectively (Figure 2A). Phylogenetic analysis showed that duDDX1 was branched with birds and showed higher evolutionary relationship than with mammals and fishes (Figure 2B).

**Table 3:** **Reference sequences information of DDX1.**

Species	GeneBank accession numbers
*HomoGallus gallus*	NP_004930.1
*Mus musculus*	NP_598801.1
*Bos taurus*	NP_001068936.1
*Ovis aries*	XP_004005743.1
*Sus scrofa*	XP_020943481.1
*Canis lupus familiaris*	XP_848865.1
*Paralichthys olivaceus*	XP_019947331.1
*Cyprinus carpio*	XP_018972473.1
*Clupea harengus*	XP_031437706.1
*Gallus*	NP_989894.1
*Anas platyrhynchos*	XP_027311055.1
*Columba livia*	XP_005506907.1
*Anser cygnoides*	XP_013028107.1
*Falco cherrug*	XP_005432942.2
*Cherry valley duck*	MT978184

### DuDDX1 expression profile in duck

**Figure 3:**
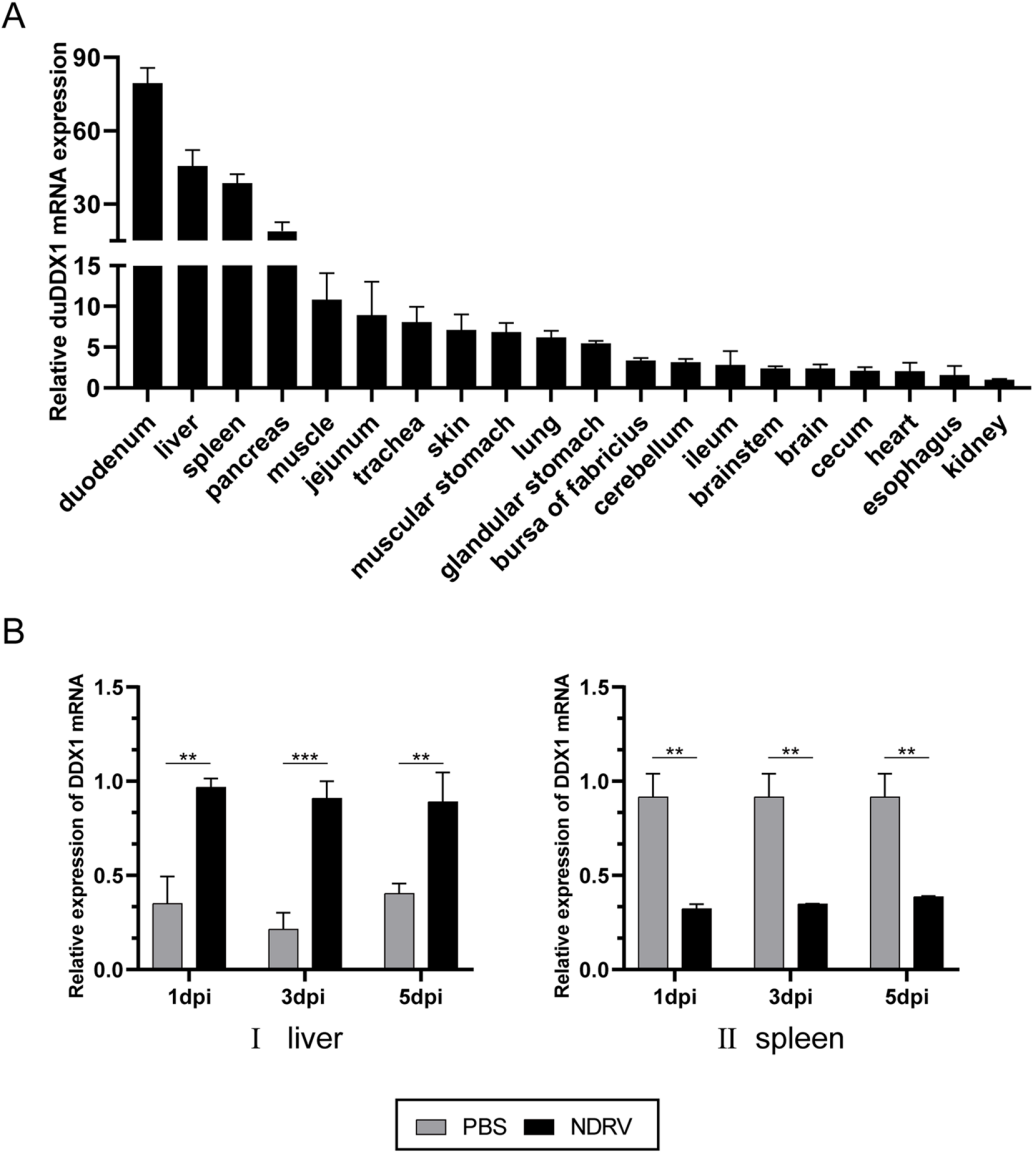
**DuDDX1 expression profile in duck.** **A** Tissue distribution of duDDX1 transcripts in healthy Cherry valley ducks. The relative mRNA levels were normalized to the expression of the GAPDH gene from various tissues. Each result represented the expression level of DDX1 relative to the kindey in the test tissue. Data are represented as the mean value ± SE of three experiments. **B** The expression level of duDDX1 in the tissues of the infected ducks. The liver (I) and spleen (II) of the experimental group (NDRV) and the control group (PBS) were collected at 1, 3, and 5 dpi after NDRV infection to detect the expression of duDDX1. A student *t* test was performed to assess the difference. ^*^P < 0.05; ^**^P < 0.01; ^***^P < 0.001.

DuDDX1 mRNA expression is relatively higher in the duodenum, liver, and spleen, while lower in the kidney and esophagus (Figure 3A). The results of animal inoculation experiments showed that after NDRV infection, the expression of duDDX1 in the liver was significantly increased 4.3-fold (*P* < 0.001), while in the spleen decreased 2.8-fold (*P* < 0.01) (Figure 3B).

#### Expression of pcDNA3.0-duDDX1-Flag in DEF cells

**Figure 4:**
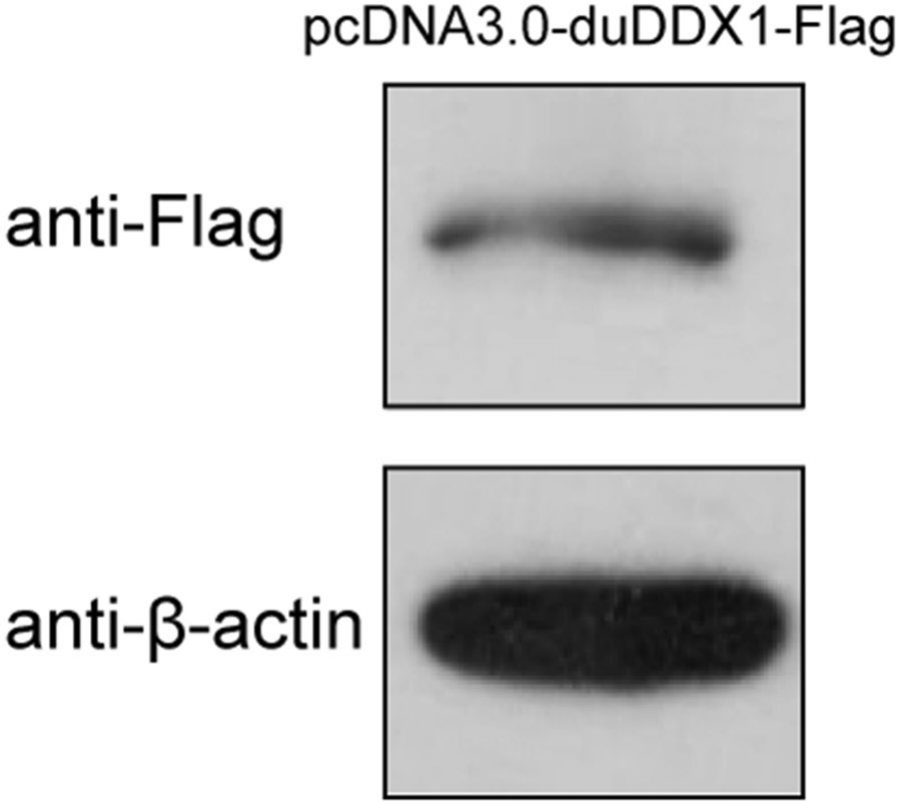
**Expression of pcDNA3.0-duDDX1-Flag in DEF cells.** Western blot detection of eukaryotic expression plasmid pcDNA3.0-duDDX1-Flag expression in DEF cells.

The western blot results showed that the band of protein expressed in DEF cells transfected with pcDNA3.0-duDDX1-Flag was about 82 KD (Figure 4), which was consistent with the size of the target protein.

### DuDDX1 is involved in innate immunity

**Figure 5:**
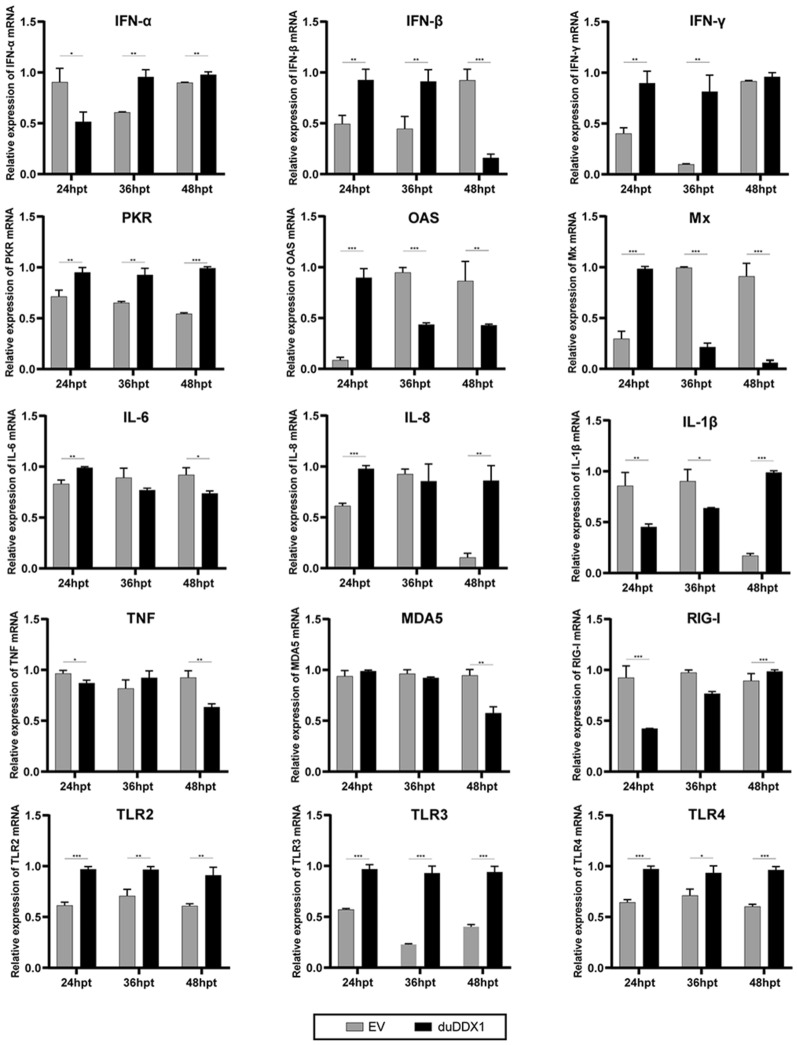
**Overexpression of duDDX1 induces gene expression of pattern recognition receptors, pro-inflammatory cytokines, interferons and anti-viral molecules in DEF cells.** The experimental group was DEF cells transfected with pcDNA3.0-duDDX1-Flag, and the control group was DEF cells transfected with empty vector. Cells were collected at 24, 36, and 48 hpt, and the mRNA expression levels of pattern recognition receptors (TLR2, TLR3, and TLR4), pro-inflammatory cytokines (IL-6, IL-8, IL-1β, and TNF), interferons (IFN-α, IFN-β, and IFN-γ) and ISGs (PKR, OAS, and Mx) were analyzed by qRT-PCR. The relative expression of gene mRNA were calculated using the 2^−ΔΔCT^ method with GAPDH serving as a normalization gene and mean control values as baseline reference. Data are represented as the mean value ± SE of three experiments. A student *t* test was performed to assess the difference. ^*^P < 0.05; ^**^P < 0.01; ^***^P < 0.001.

To determine the role of duDDX1 in duck innate immunity response, the pcDNA3.0-duDDX1-Flag or empty vector were transfected into DEF cells, and DEF cells were collected at designated time points for subsequent experiments (Figure 5). Figure 5 shows that the mRNA expression levels of Toll-like receptors (TLR2, TLR3, and TLR4) were up-regulated after 12 hpt versus the control group. The mRNA expression level of TLR3 was up-regulated by 4.1-fold (*P* < 0.001) at 36 hpt. In addition, the mRNA expression levels of IFN-β and IFN-γ were up-regulated both at 12 and 24 hpt versus the control group. The mRNA expression level of IFN-γ was up-regulated by 8.3-fold (*P* < 0.01) at 36 hpt.

### The NDRV induced DuDDX1 participates in the immune response by mediating IFN-β

**Figure 6:**
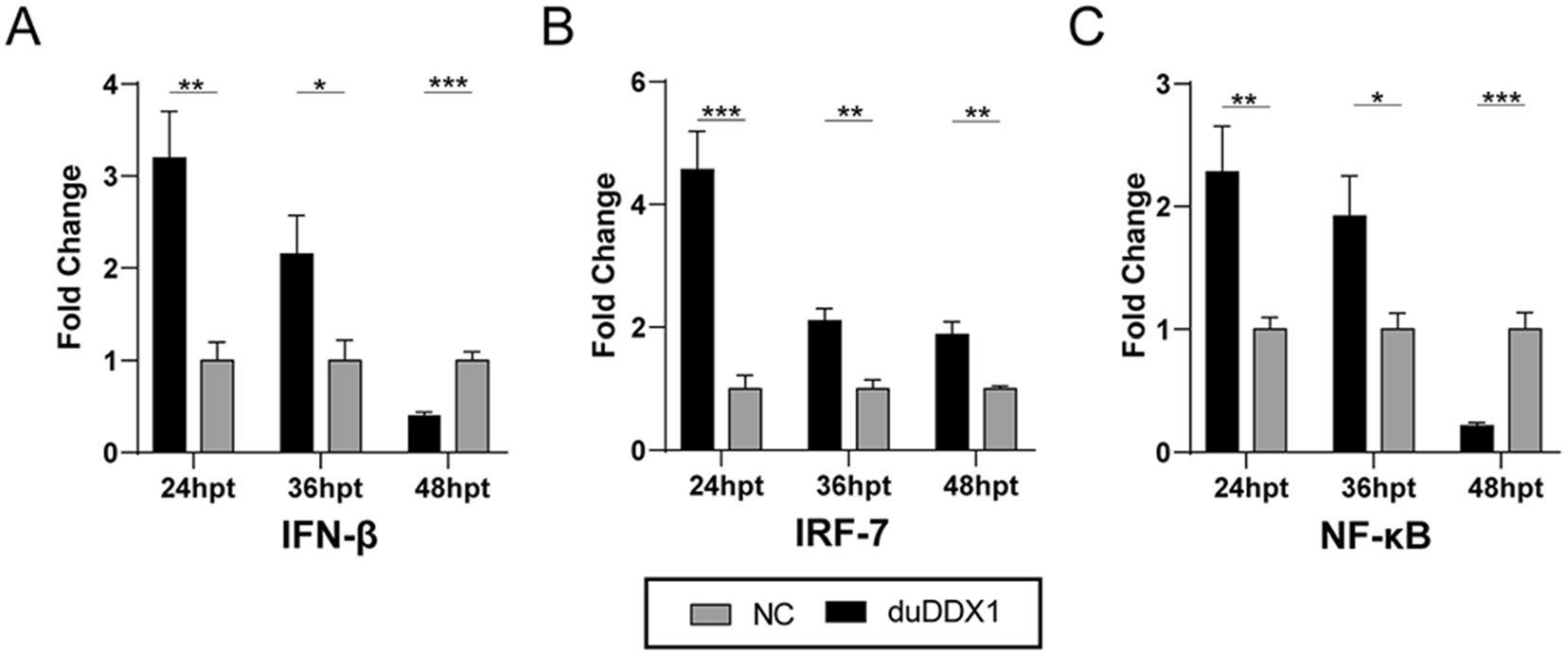
**Overexpression of duDDX1 stimulates IFN-β expression.** **A** dual luciferase reporter gene assay was used to study the IFN-β signaling pathway. PcDNA3.0-duDDX1-Flag or empty vector (500 ng/well) were co-transfected with reporter plasmids (100 ng/well) (**A**) pGL3-IFN-β; (**B**) pGL3-IRF-7; (**C**) pGL-NF-κB with pRL-TK (normalization) (50 ng/well). After 24, 36, and 48 hpt, cells were harvested, and luciferase activity was measured. Relative IFN-β-, IRF-7-, or NF-κB-reporter activation was calculated as fold-change in normalized Firefly luciferase activity with reference to mean control values set to 1. Data were means from three independent experiments and each experiment was analyzed in tri plicate. A student *t* test was performed to assess the difference. ^*^ *P* < 0.05; ^**^ *P* < 0.01; ^***^ *P* < 0.001.

To further demonstrate that duDDX1 is involved in the signaling pathway of IFN-β in DEF cells, we conducted the dual luciferase experiment (Figure 6). The results showed that duDDX1 significantly activated the activity of IFN-β (3.2-fold, *P* < 0.01), and both IRF-7 and NF-κB promoters were involved in regulating duDDX1-induced IFN-β activation (4.5-fold, *P* < 0.001).

### DuDDX1 has antiviral activity

**Figure 7:**
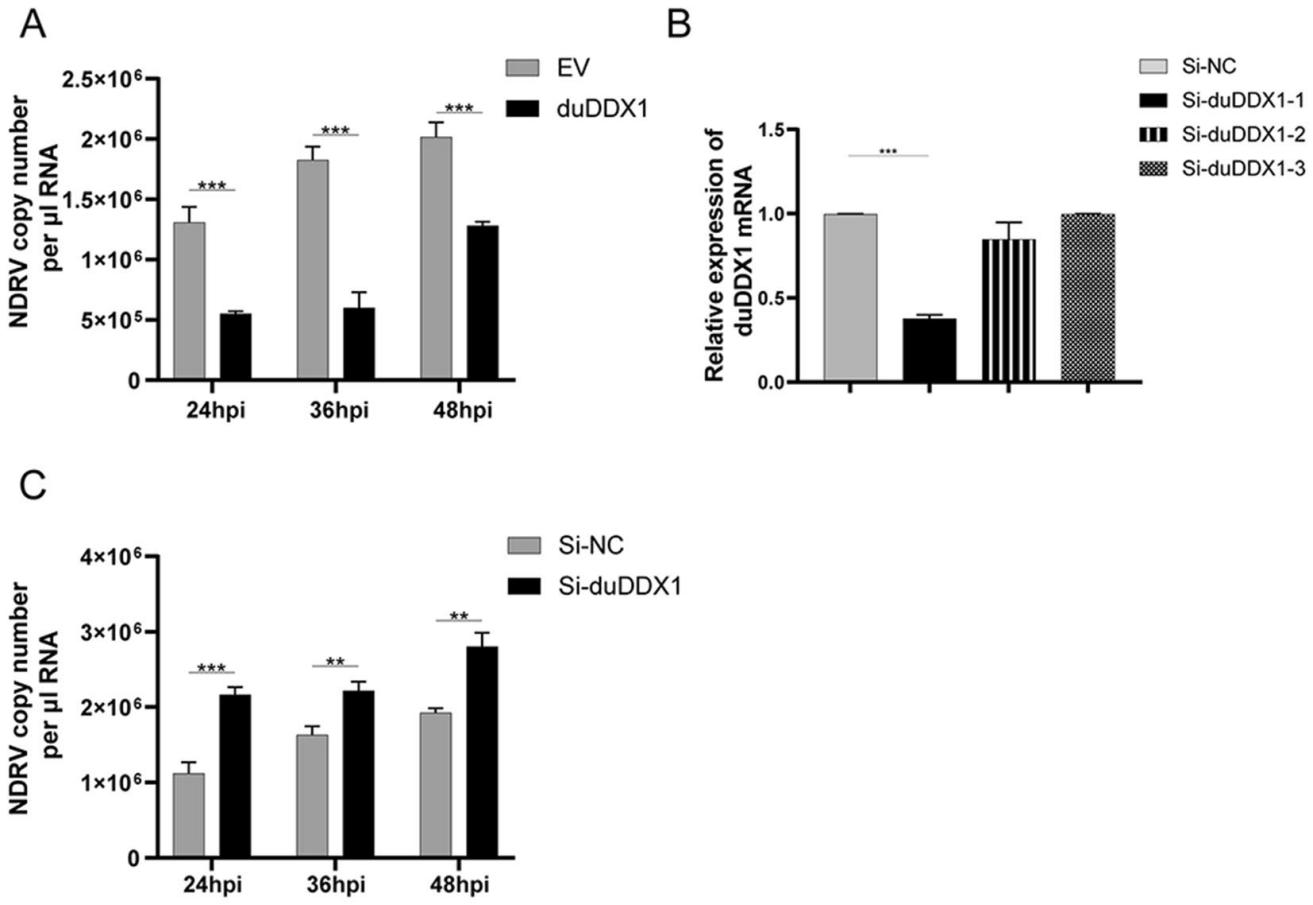
**DuDDX1 inhibits NDRV virus activity.** **A** Transfected pcDNA3.0-duDDX1-Flag or empty vector in DEF cells for 24 h and then the cells were infected with 10 TCID_50_/mL NDRV. **B** SiRNA interference efficiency. DEF cells were transfected with Si-duDDX1-1 as the experimental group or transfected with Si-NC as the control group. The cells were collected at 36 hpt. QRT-PCR was used to detect the expression level of duDDX1 mRNA. **C** Transfected Si-duDDX1 or Si-NC in DEF cells for 36 h and then the cells were infected with 1 TCID_50_/mL NDRV. The culture supernatant (**A**, **B** are the same) were collected, and detected the virus titer at 24, 36, and 48 hpi by qRT-PCR. A student *t* test was performed to assess the difference. ^*^P < 0.05; ^**^P < 0.01; ^***^P < 0.001.

To analyze the effect of DDX1 on the proliferation of NDRV, DEF cells transfected with pcDNA3.0-duDDX1-Flag or empty vector were infected with NDRV. The changes of viral RNA were measured by qRT-PCR to confirm the antiviral function of duDDX1 (Figures 7A, C). Figure 7A shows that compared with the control group, the copy number of NDRV at 36 h post-infection (hpi) was reduced by 3.0-fold (*P* < 0.001). To verify this result, three interfering RNAs of duDDX1 were designed (Figure 7B). Figure 7B shows that Si-duDDX1-1 displayed the highest interference efficiency. Therefore, Si-duDDX1-1 was selected as the interfering RNA verify the above experimental results. The results showed that the inhibition of duDDX1 by siRNA interference showed increased NDRV replication (Figure 7C).

### DuDDX1 impacts antiviral and innate immune responses after NDRV infection

After overexpression or interference with duDDX1, DEF cells were stimulated by NDRV to investigate the changes in antiviral and innate immune response after NDRV infection.

**Figure 8:**
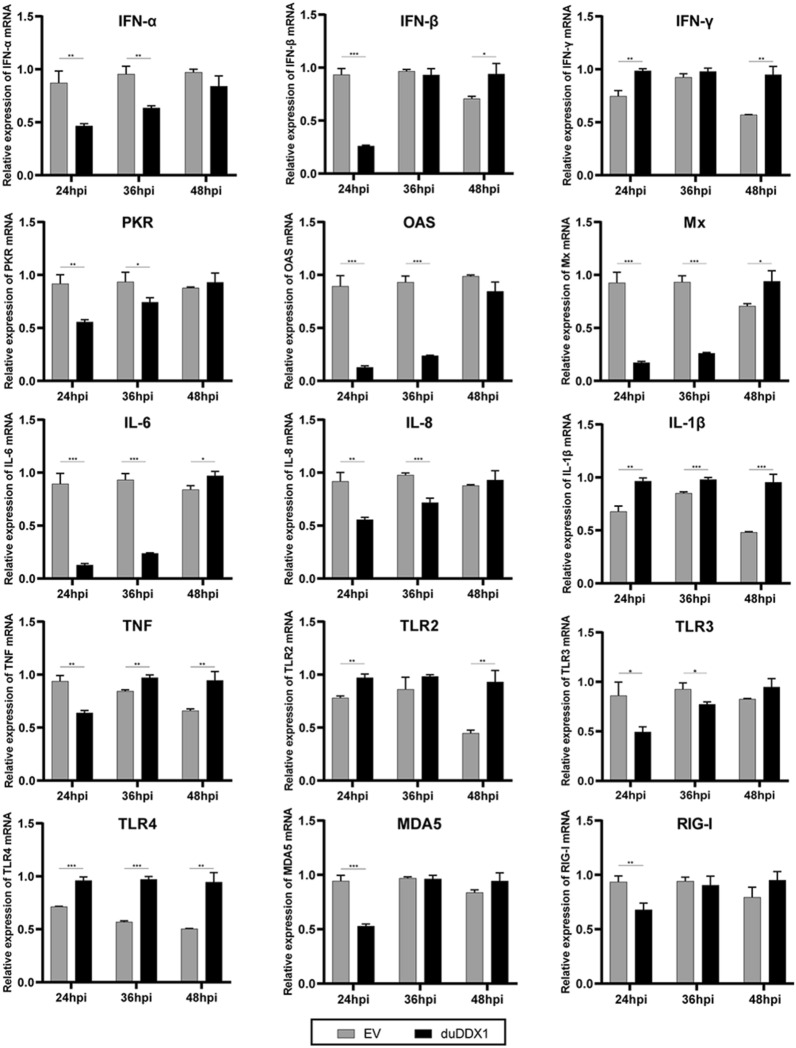
**DuDDX1 over-expression in DEF cells modulates gene expression pattern of pattern recognition receptors, cytokines and anti-viral molecules after NDRV infection.** DEF cells were transfected with pcDNA3.0-duDDX1-Flag as the experimental group, and DEF cells were transfected with empty vector as the control group. The cells were stimulated with 10 TCID_50_/mL NDRV after 24 hpt. At 24, 36, and 48 hpi, cells were collected and analyzed for inducible gene expression. The relative expression of gene mRNA were calculated using the 2^−ΔΔCT^ method with GAPDH serving as a normalization gene and mean control values as baseline reference. Data are represented as the mean value ± SE of three experiments. A student *t* test was performed to assess the difference. ^*^P < 0.05; ^**^P < 0.01; ^***^P < 0.001.

We found that the Toll-like receptors (TLR2 and TLR4) involved in the antiviral response were up-regulated after viral stimulation (2.1-fold, *P* < 0.01), while TLR3 was down-regulated 1.7-fold (*P* < 0.05). The expression levels of OAS and Mx mRNA were down-regulated by 7.0-fold (*P* < 0.001) and 5.4-fold (*P* < 0.001) at 24 hpi, respectively (Figure 8).

**Figure 9:**
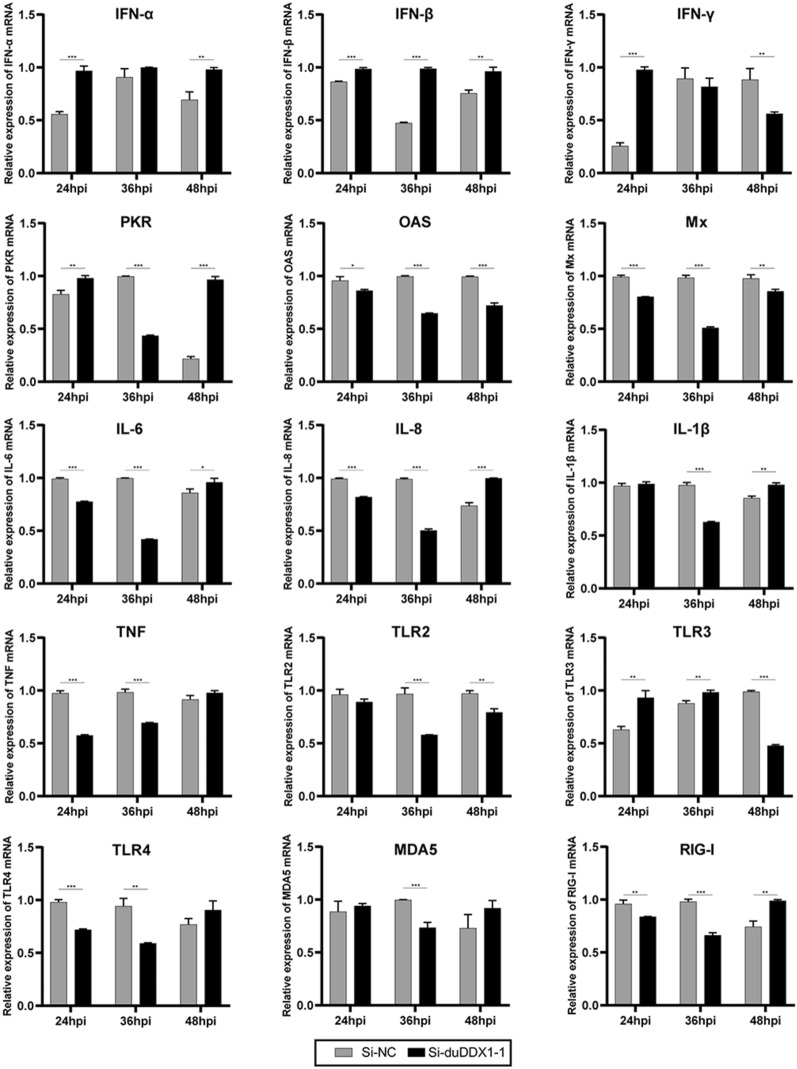
**Knocking-down duDDX1 expression reduces induction of some major innate immune and anti-viral gene expression after NDRV infection.** DEF cells were transfected with Si-duDDX1-1 as the experimental group, and DEF cells were transfected with Si-NC as the control group. The cells were stimulated with 1 TCID_50_/mL NDRV after 36 hpt. Cells were collected and analyzed for inducible gene expression at 24, 36, and 48 hpi. The relative expression of gene mRNA were calculated using the 2^−ΔΔCT^ method with GAPDH serving as a normalization gene and mean control values as baseline reference. Data are represented as the mean value ± SE of three experiments. A student *t* test was performed to assess the difference. ^*^P < 0.05; ^**^P < 0.01; ^***^P < 0.001.

Figure 9 shows that after virus stimulation, IFN-α, IFN-β, and IFN-γ were all up-regulated, and IFN-γ was up-regulated by 3.8-fold (*P* < 0.001) at 24 hpi. In addition, at 24 and 36 hpi, TLR2 and TLR4 were down-regulated, while TLR3 was up-regulated. At 48 hpi, PKR was up-regulated by 4.5-fold (*P* < 0.01). Gene knockdown is roughly the opposite of gene overexpression (Figure 9).

## Discussion

In this research, the complete DDX1 cDNA was cloned from Cherry valley duck for the first time. DuDDX1 encoded an amino-acid sequence of 740 residues. Based on the predicted amino acid sequence, bioinformatics analysis was performed. Like DDX1 of other species, duDDX1 contains three main domains: DEXDc domain (AA 21–444), HELICc domain (AA 520–610), and SPRY domain (AA 130–246). Multiple sequence alignment revealed that the amino sequence of duDDX1 was highly conserved among various species including mammals, birds and fishes. To investigate the tissue-specific distribution of duDDX1, the abundance of duDDX1 gene in different tissues was analyzed. DuDDX1 mRNA was detected in all test tissues. DuDDX1 is highly expressed in the duodenum, liver, and spleen. However, porcine DDX1 is abundantly expressed in fat, spleen and liver. These results indicate that the distribution of DDX1 in different species is different. Western blot results show that the pcDNA3.0-duDDX1-Flag eukaryotic expression plasmid was successfully constructed and can be expressed in DEF cells.

To further explore the relationship between duDDX1 and host innate immunity, we found that overexpression duDDX1 in DEF cells up-regulated TLRs (TLR2, TLR3, and TLR4) and IFN-I (IFN-α, IFN-β, and IFN-γ), however, some cytokines (IL-6, IL-8, IL-1β, and TNF) and RIG-I-like receptors (RIG-I and MDA5) did not change significantly.

In the host's innate immunity, type I IFN is a key cytokine, which can induce a large number of interferon-stimulating genes against viruses [30]. Previous studies have confirmed that DDX1 can promote the production of type I IFN. According to reports, the RNA helicase complex composed of DDX1, DDX21 and DHX36 induces type I IFN through TRIF-dependent signaling in myeloid dendritic cells, and they can recognize short poly I: C (0.2–1 kb) and long poly I: C (1.5–8 kb) and reovirus [31, 32]. The non-structural protein 14 (nsp 14) encoded by Transmissible gastroenteritis virus (TGEV) interacts with DDX1 to induce the production of IFN-β [33]. We found that overexpression of duDDX1 can significantly activate the activity of IFN-β, and both IRF-7 and NF-κB promoters are involved in regulating duDDX1-induced IFN-β activation. This suggests that duDDX1 may be involved in inducing the expression of IFN-β.

In general, viral infection triggers the host's innate immune response through activation of PRRs [34]. DDX1 can interact with the nsp 14 protein from severe acute respiratory syndrome coronavirus (SARS-CoV) to enhance Avian infectious bronchitis virus (IBV) replication [35]. In addition, DDX1 can promote the proliferation of hepatitis C virus (HCV) [19]. DDX1 can also interact with the viral protein 3D and inhibit the replication of the foot-and-mouth disease virus [36]. Although DDX1 can inhibit or promote virus replication, however, the role of DDX1 during NDRV infection remains unclear. Our results showed that the replication of NDRV was significantly inhibited in DEF cells overexpressing duDDX1, while it was enhanced in DEF cells knocking down duDDX1, it can be concluded that duDDX1 can inhibit the proliferation of NDRV in vitro. These changes have a certain relationship with the natural immunity caused by the host infection with NDRV virus.

In this study, we cloned the full length CDs of duDDX1, to further study the role of duDDX1 in the host's innate immune response, Cherry valley ducks were infected with NDRV. The expression level of duDDX1 mRNA was up-regulated in the liver, but down-regulated in the spleen. This indicates that DDX1 may be involved in the anti-NDRV immune response, then we further verified it through in vitro cell experiments. By comparing the effects of overexpression or knockdown of duDDX1 on the immune response in DEF cells infected with NDRV, we found that after infection with NDRV virus, the expression of inflammatory cytokines decreased and the expression of TLRs changed significantly, except RIG-I receptors, we speculate that duDDX1 may regulate the expression of TLRs, thereby regulating the expression of interferons and viral stimulating factors. According to reports, NDRV can be recognized by several PRRs that initiate innate immunity [29], which is similar to our results. As we concluded that duDDX1 can inhibit the replication of NDRV virus.

In summary, NDRV has caused a huge economic losses to the breeding industry [8], therefore, investigating the immune mechanism of duDDX1 defense against NDRV infection will provide a theoretical basis for further understanding of the antiviral innate immune response of ducks and the pathogenesis of inflammatory diseases.

## Data Availability

We have uploaded the acquired sequence of duDDX1 to GenBank (accession number, MT978184), and other datasets analyzed during the current study are available from the corresponding author on reasonable request.

## Abbreviations

DDX1: DEAD-box helicase 1
TLRs: Toll-like receptors
duDDX1: Duck DDX1
DEF: Duck embryo fibroblast
NDRV: Novel duck reovirus
TCID_50_: 50% Tissue culture infective dose
PVDF: Polyvinylidene fluoride
FITC: Fluorescein isothiocyanate
qRT-PCR: Quantitative real-time PCR
hpt: Hours post-transfection
hpi: Hours post-infection
SE: Standard error
EV: Empty vector
